# Isolated Laryngeal Angioedema in a Patient with Long-term ACE Inhibitor Use: A Case Report

**DOI:** 10.5811/cpcem.1565

**Published:** 2024-01-23

**Authors:** Carney Flinn, Inna Massaro

**Affiliations:** * Virginia Tech Carilion School of Medicine, Roanoke, Virginia; † Roanoke Memorial Hospital, Department of Emergency Medicine, Carilion Health System, Roanoke, Virginia

**Keywords:** *angioedema*, *laryngeal edema*, *ACE inhibitor*, *case report*

## Abstract

**Introduction:**

Angiotensin converting enzyme (ACE) inhibitor-associated angioedema is the most common cause of angioedema seen in the emergency department (ED) and can be associated with a high morbidity. Most cases occur within months of initiation of an ACE inhibitor and are associated with facial and/or oropharyngeal swelling. We present a case of isolated laryngeal edema requiring intubation following 10 years of ACE inhibitor therapy.

**Case Report:**

An 82-year-old female, who was on lisinopril therapy for 10 years, presented to the ED with shortness of breath and a sensation that her throat was swelling. She appeared to be in mild respiratory distress and could only speak in one-word sentences. On the physical exam, there was no swelling in the tongue, lips, or face, and the uvula was midline. There was mild posterior pharyngeal edema and swelling noted, but the airway was not visibly obstructed. She was tachypneic and stridor was present. After no improvement with medications, anesthesia successfully intubated her in the operating room. It was deemed a difficult airway secondary to posterior pharyngeal erythema and edema. She was diagnosed with ACE inhibitor-associated angioedema and was extubated four days later. Her lisinopril was discontinued, and she has not had a recurrence of angioedema.

**Conclusion:**

ACE inhibitor-induced angioedema commonly presents with facial and oropharyngeal swelling. Its recognition, even years after starting an ACE inhibitor, is necessary to ensure swift and appropriate treatment of potentially life-threatening posterior pharyngeal edema.

Population Health Research Capsule What do we already know about this clinical entity?

*Angiotensin converting enzyme (ACE) inhibitor-associated angioedema (AE) often occurs in initial months and is associated with facial and/or oropharyngeal swelling.*

What makes this presentation of disease reportable?

*In this atypical presentation, isolated laryngeal AE required intubation after 10 years of ACE inhibitor therapy.*

What is the major learning point?

*Recognizing AE, even years after a patient has started therapy, is necessary to ensure appropriate treatment of potential life-threatening posterior pharyngeal edema.*

How might this improve emergency medicine practice?

*ACE inhibitor-associated AE is the most common cause of drug-induced AE and can have high morbidity. Awareness of unusual presentations may be life-saving.*

## INTRODUCTION

Angioedema (AE), defined by non-dependent, non-pitting edema, is a potentially life-threatening condition, complicated by a compromised airway due to laryngeal edema. There are two main types: histamine mediated, and bradykinin mediated. In all types of AE, facial swelling, including the lips and the oral mucous membranes, is common. Histamine-mediated forms present similarly to anaphylaxis while the bradykinin-mediated forms typically present with greater face and oropharyngeal involvement with increased risk for progression and morbidity.^1^ ^,^ ^2^ ^,^ ^3^ ^,^ ^4^ ^,^ ^5^

Angiotensin converting enzyme (ACE) inhibitors are the leading cause of drug-induced AE and commonly presents with swelling of the lips, tongue, or face.^6^ ^,^ ^7^ One study found 100% of participants with ACE inhibitor-related AE reported facial swelling.^8^ Swelling of the larynx, which has the potential to develop rapidly with life-threatening implications, was highest in ACE inhibitor-induced AE.^8^ Most cases typically occur within the first three months of therapy, although there is a recognized persistent risk of AE in patients taking an ACE inhibitor despite an uneventful initiation of treatment.^2^ ^,^ ^9^ ^,^ ^10^ ^,^ ^11^

In previously documented cases of AE occurring after years of stable therapy, patients typically presented with facial and oropharyngeal swelling,^6^ ^,^ ^7^ ^,^ ^9^ although one case report in the internal medicine literature describes isolated posterior pharyngeal and supraglottic swelling while on ACE inhibitor therapy for one year.^12^ The far more common AE presentation is facial and oropharyngeal swelling after one year of ACE inhibitor use.^11^ ^,^ ^12^ We present a case of isolated laryngeal AE following 10 years of ACE inhibitor therapy. This case features an atypical presentation of bradykinin-mediated AE and highlights the importance of a high index of suspicion and securing/maintaining an airway in patients with possible AE.

## CASE REPORT

An 82-year-old female presented to the emergency department (ED) with a chief complaint of throat swelling and difficulty breathing with a past medical history including chronic obstructive pulmonary disease, lung cancer, congestive heart failure, dyslipidemia, diabetes mellitus type two, gastroesophageal reflux disease, hypertension, and hypothyroidism. Her outpatient medications included lisinopril 20 milligrams (mg), aspirin 81 mg, atenolol 25 mg, budesonide-formoterol 160–4.5 micrograms (mcg), hydrochlorothiazide 25 mg, insulin glargine 100 Units (U) per milliliter (mL), levothyroxine 100 mcg, metformin 500 mg, omeprazole 20 mg, and simvastatin 20 mg. The patient stated she awoke the same morning with an inability to swallow water or her own saliva because it felt like her throat was swelling. She’d had a very mild cough for the prior few days and a sore throat the night before, which resolved with acetaminophen. She stated she felt well before going to bed. She denied a history of anaphylaxis or allergic reactions.

On review of systems, the patient denied nausea, vomiting, fevers, or chills. Her vitals included a temperature of 36° Celsius, respiratory rate of 26 breaths per minute, oxygen saturation of 95% on room air, pulse rate of 93 beats per minute, and a blood pressure of 119/56 millimeters of mercury. Physical exam revealed the patient was alert, age-appropriate, and markedly anxious. She was spitting into a bag and speaking in single-word sentences. Her voice was not hoarse. Mild posterior pharyngeal edema and swelling was noted. The airway was not visibly obstructed; there was no tongue or lip swelling, and the uvula was midline and not enlarged. Her cardiovascular exam displayed a regular rate and rhythm. She was tachypneic, but breath sounds were normal in all lung fields; however, stridor was present. Her exam was otherwise unremarkable.

Lab values were as follows: complete blood count was within normal limits except for mild thrombocytopenia, complete metabolic panel showed an elevated carbon dioxide of 32 millimoles per liter (mmol/L) (reference range 21–31 mmol/L), elevated creatinine of 1.22 mg per deciliter (dL) (0.5–1.2 mg/dL), elevated urea nitrogen of 23 mg/dL (6–20 mg/dL), and elevated blood glucose of 181 mg/dL (70–99 mg/dL). Additionally, her magnesium was low at 1.0 mg/dL (1.7–2.8 mg/dL). All other lab values including a venous blood gas, troponin, brain natriuretic peptide, creatine kinase. and lactate dehydrogenase were within normal levels. After nasal swabbing, no respiratory syncytial virus, influenza, adenovirus, human metapneumovirus, or rhinovirus were detected. No imaging was done initially.

Medications administered included racemic epinephrine 2.25% nebulized solution, methylprednisolone 125 mg intravenous (IV), famotidine 20 mg IV, and diphenhydramine 25 mg IV. Intramuscular epinephrine was withheld secondary to patient’s coronary artery disease. Her symptomology did not improve following administration of medications, and her shortness of breath continued to worsen. The patient had been taking lisinopril for 10 years, and there was a strong clinical suspicion for upper airway compromise. After consult with general surgery and anesthesia, she was taken electively to the operating room (OR) for intubation.

The anesthesia team successfully intubated her after two attempts with a 7–0 endotracheal tube after bougie exchange. It was deemed a difficult airway secondary to posterior pharyngeal edema. The epiglottis did not appear to be inflamed, but there was a pooling of secretions in the posterior pharynx. She was assigned an American Society of Anesthesiologists physical status classification of 4. The patient was successfully extubated four days later after the swelling subsided. Her lisinopril was discontinued, and to date she has not had a recurrence of AE.

## DISCUSSION

Angioedema is a potentially life-threatening condition characterized by non-pitting, non-dependent edema. Angioedema can be either histamine-mediated, which presents similar to an allergic reaction, or bradykinin-mediated.^1^ ^,^ ^2^ The bradykinin-mediated types include a rare disorder called hereditary angioedema in which patients lack complement 1 inhibitor (C1-INH), leading to excessive production of bradykinin and an increased risk for AE attacks.^1^ ^,^ ^13^ A second, more common cause of bradykinin-mediated AE is ACE inhibitors. It is well established that ACE inhibitors, which are widely used to treat hypertension, congestive heart failure, and diabetic nephropathy, lead to a buildup of bradykinin.

ACE inhibitor-associated AE is one of the most common causes of AE seen in the ED and can be associated with a high morbidity. Presentation can range from mild facial edema to acute laryngeal or subglottic involvement, which could prove life-threatening. Cases commonly present with swelling of the face (52%), lips (49%), or tongue (approximately 20%). While symptoms typically present within four weeks of starting therapy, they have been documented to occur after years of stable therapy.^6^ ^,^ ^7^ ^,^ ^14^ Cases of acute facial and airway edema due to ACE inhibitors may be misdiagnosed as an anaphylactic reaction, and the association with ACE inhibitors may be ignored or missed; thus, it is important to distinguish between the two.^4^ ^,^ ^9^ ^,^ ^15^ Atypical presentations such as difficulty swallowing (9%), difficulty speaking (6%), stridor/dyspnea (5%), hoarseness (3%), and increased salivation and difficulty handling oral secretions (2%) may cause a delay of treatment and potential increase in morbidity.^14^ As airway compromise is not uncommon among patients with ACE inhibitor-associated AE, it is critical to have early recognition and management.^12^

Management of ACE inhibitor-associated AE in the ED includes assessment of the airway. Medications rarely alleviate the swelling, unlike in histamine-mediated AE. Angioedema can progress rapidly, and for patients who require definitive airway management, cricothyrotomy or tracheostomy is needed in up to 50% of cases.^4^ The presence of epiglottic or laryngeal edema suggests the need for a definitive airway. If the swelling is restricted to the structures anterior to the teeth, such as the lips, intubation is generally not needed. Noninvasive positive pressure ventilation is not a definitive therapy for patients with airway involvement. Additionally, supraglottic and extraglottic airway devices are not recommended in patients with AE, as it will not secure an airway below the site of obstruction and can worsen edema due to trauma.

Severe edema may prohibit passage of an endotracheal tube through the glottis, even with the use of fiberoptic or video laryngoscopy guidance; therefore, the resuscitation team should be prepared for cricothyrotomy before an attempt at intubation is started. If the patient does not require immediate airway intervention, it may be beneficial to consult anesthesia and otolaryngology and consider transferring the patient to the OR.^4^ ^,^ ^12^ Ultimately, treatment for ACE inhibitor-associated AE is to stop use of the ACE inhibitor and for the patient to consider an alternate medication with their primary care physician.

## CONCLUSION

An elderly female who had been using an ACE inhibitor for 10 years presented to the ED with a chief complaint of throat swelling and difficulty breathing. Recognition of angioedema is necessary to ensure swift and appropriate treatment. There should be a low threshold to treat and a high suspicion of AE, even years after initial treatment with ACE inhibitors.
